# Evaluation of Peri-Implantitis through Fourier-Transform Infrared Spectroscopy on Saliva

**DOI:** 10.1021/acs.jproteome.4c00707

**Published:** 2025-01-10

**Authors:** Graziele Parize, Gabrielle Luana Jimenez, Jamil Awad Shibli, Rafael Siroma, Matheus Willian Caetano, Yeon Jung Kim, Paulo Henrique Braz-Silva, Herculano da Silva Martinho, Debora Pallos

**Affiliations:** † University of Santo Amaro (UNISA), Rua Isabel Schmidt 349, São Paulo 04743-030, Brazil; ‡ Centro de Ciências Naturais e Humanas, Universidade Federal Do ABC (UFABC), Santo André, São Paulo 09280-560, Brazil; § Department of Periodontology, Dental Research Division, Guarulhos Univeristy, Guarulhos, São Paulo 07023-070, Brazil; ∥ Department of Stomatology, School of Dentistry, University of São Paulo, Sao Paulo 05508-000, Brazil; ⊥ Laboratory of Virology (LIM-52-HCFMUSP), Institute of Tropical Medicine of São Paulo, University of São Paulo School of Medicine, São Paulo 05403-000, Brazil

**Keywords:** peri-implantitis, Fourier transform infrared spectroscopy, attenuated total reflectance, liquid biopsy, biophotonics, saliva, diagnosis

## Abstract

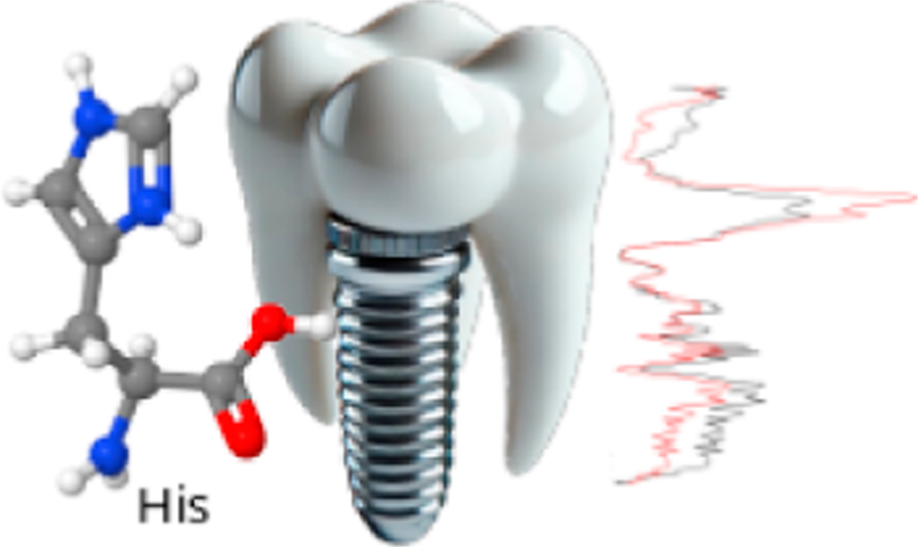

Background: Peri-implantitis is characterized as a pathological change in the tissues around dental implants. Fourier-transform infrared spectroscopy (FTIR) provides molecular information from optical phenomena observed by the vibration of molecules, which is used in biological studies to characterize changes and serves as a form of diagnosis. Aims: this case–control study evaluated the peri-implant disease by using FTIR spectroscopy with attenuated total reflectance in the fingerprint region. Methods: 38 saliva samples were evaluated, 17 from the control group and 21 from the peri-implantitis group. Clinical data such as plaque index (PI), gingival index, probing depth (PS), and attachment level were assessed. Results: The results of clinical parameters showed a statistical difference between the two groups regarding an excess of the PI. In the FTIR-ATR analysis, the main components revealed vibrational modes of fatty acids, histidine, lipid esters, nucleic acids, and tryptophan, with the main molecules contributing to spectral discrimination. The five-component partial least-squares discriminant analysis classification model had an accuracy of 81%, showing differences between healthy and diseased implants. Conclusion: the FTIR spectroscopy provides important molecular characteristics of the samples and the results in association with clinical data show the effectiveness of using this tool for diagnosing the disease.

## 1 Introduction

Saliva is composed of water (99%) and a variety of inorganic ions such as potassium (K^+2^), calcium (Ca^+^), magnesium (Mg^2+^), and proteins (i.e., immunoglobulins, urea, uric acid), which make up the remaining portion.^1^ More than 700 microorganisms have also been found in the oral cavity, which may be related to oral health and other periodontal and systemic diseases.^2^ It has been suggested that the human oral microbiome contributes to more than 2000 microbial proteins from more than 50 bacterial *genera*.^1,3^

Salivary biomarkers have been suggested as suitable for regular screening and early detection of many conditions, such as some types of cancers (e.g., ovary, pancreas, breast, and mouth) and some degrees of dementia (e.g., Alzheimer’s disease).^4,5^

In recent decades, the placement of dental implants has become a routine procedure in oral rehabilitation. However, the number of patients and patients affected by peri-implant diseases is increasing. Since there are no established and predictable concepts for the treatment of peri-implantitis, primary prevention is of fundamental importance. Early diagnosis and management of mucositis are considered preventive measures against the onset of peri-implantitis.^6^

Spectroscopic techniques have emerged as one of the main tools for biomedical application, being increasingly used in the field of clinical evaluation and diagnosis.^7^ Fourier-transform infrared (FTIR) spectroscopy works by measuring the absorption of infrared radiation.^8^ It is a vibrational spectroscopic technique used to probe molecular changes associated with tissues. The method is used in more conservative analyses of the characteristics within tissues and cells, including attributions of functional groups, types of bonds, and molecular conformations.^7^ This technique is particularly attractive for analysis of biofluids as it is easy to use, requires little sample preparation, and is adaptable to various body fluids, which reduces the time of analysis and allows results to be obtained quickly.^9^ FTIR is relatively simple, reproducible, nondestructive to tissue and requires small amounts of material (from micrograms to nanograms), meaning that minimal sample preparation is needed for analysis. The spectral bands in vibrational spectra are molecule-specific and provide direct information on their composition, serving as a tool for disease detection and monitoring.^7,10^

Thus, the main objective of this investigation is probing possible identifiers by FTIR salivary profiles that show relevant alterations and correlate this finding to peri-implant disease.

## 2 Materials and Methods

### 2.1 Demographic Data

The study was approved by the Ethics Committee of the University of Guarulhos according to protocol no. UNG ID 205/03 and carried out in accordance with the Helsinki Declaration. The study protocol was explained to each participant, who signed an informed consent form. This is a case-control study based on previous studies already published by our group.^11−13^ For the case-control study, the STROBE guidelines were followed.

Saliva samples were collected in sterile containers in the morning as described elsewhere.^12,13^ Briefly, all patients had been fasting for more than 8 h, and no stimulation was required for saliva collection. A minimum of 3 mL of saliva was requested from each patient and immediately stored in cryogenic tubes, properly identified, numbered, and kept refrigerated in liquid nitrogen during transport. Next, they were stored in an ultrafreezer (−80 °C) at the Federal University of ABC, Santo André campus, until analysis.

Forty patients of both genders were included in the study and divided into two groups: those with healthy implants (Group C) and those with peri-implantitis (Group P).

For defining peri-implantitis, we used the Berglundh et al., 2017 classification that outlines specific diagnostic criteria, including the presence of bleeding and/or suppuration upon gentle probing, probing depths of ≥6 mm, and bone levels ≥3 mm apical to the most coronal portion of the intraosseous part of the implant.^11,14^

Inclusion criteria were healthy patients above 18 years old presenting no systemic impairment and having at least one single osseointegrated implant in function for at least two years.

Exclusion criteria were patients under 18 years old, if they had implants with coated surfaces, moderate to severe chronic periodontitis characterized by suppuration, bleeding on probing in more than 30% of subgingival sites, or any site with a probing depth (PD ≥ 5 mm), had taken antibiotics or anti-inflammatory drugs within 6 months prior to the clinical examination, had undergone periodontal or peri-implant therapy within the last 6 months, had a chronic medical disease or condition, had implant-supported prostheses with mobile abutments and/or screws, or fractured prosthetic crowns made of ceramic or resin (to avoid occlusal interference), had clinically detectable implant mobility (indicating a lack of osseointegration), or were smokers.

### 2.2 Clinical Parameters

Investigator calibration was performed as previously described,^11^ and the standard error of measurement was calculated. The interexaminer variability was 0.25 mm for PD and 0.3 mm for attachment level (AL).

Data from each patient of the control and peri-implantitis groups were collected, including gender and age. Plaque index (PI), gingival index (GI), PD, and AL were obtained from six sites of each implant (i.e., mesio-buccal, buccal, disto-buccal, disto-lingual, lingual and mesio-lingual) and evaluated by a calibrated examiner.^11^ The measurements were made by using a North Carolina periodontal probe (PCPNU-15, Hu-Friedy, Chicago, IL, USA).

### 2.3 Sample Preparation for FTIR Measurements

Sample analyses were performed according to the protocol patented by one of the authors under number 29409161929424514/INPI and described on Teodoro Napomuceno et al.^15^ The drying methodology consisted of dropping 1 μL of raw saliva onto a platinum substrate under controlled temperature and humidity conditions. Several parameters were analyzed to ensure reproducibility, including drying patterns, sample dilution, drying temperature, and relative humidity during the drying time. Some dilutions were tested as described in the aforementioned patent and reference.

The literature on biofluids indicates that a relative humidity of 80% is the ideal drying condition for creating the ideal environment, in which the saliva is treated with NaCl (sodium chloride) solution to avoid a coffee-ring effect and obtain a homogeneous biofilm. Next, the treated saliva was placed inside a desiccator and kept at equilibrium for 24 h to maintain the relative humidity of 80%. The room temperature was controlled at 20 °C.

After stabilization, the samples were taken out of the ultrafreezer and thawed at room temperature of 25 °C before pipetting 1 μL of saliva onto the substrates. The drops were prepared in triplicate. The saliva already deposited on the substrate was then placed in the desiccator for the drying process under controlled humidity for 24 h. The ideal substrates must be mechanically resistant, allowing good reflection images under an optical microscope without oxidizing under the action of the saliva constituents; hence, the choice of hard disks, as they contain platinum in their composition.

The platinum substrates were taken for visual inspection under an optical microscope at the Central Experimental Multiuser Laboratory of the Federal University of ABC (UFABC), but the samples were not effectively dried for this analysis with a microscope. Therefore, it was decided to perform the analysis using the attenuated total reflectance (ATR) technique to obtain better results and greater reliability. The micro-ATR technique is an ideal complement to FTIR spectroscopy for in situ real-time analysis and monitoring of chemical reactions. The reaction mixtures are typically dense and optically thick to mid-infrared radiation. The limited penetration depth of infrared energy into the reaction solution in contact with the ATR sensor allows for the recording of high-quality FTIR spectra.^8,16^ ATR analysis is performed with diamond tip and helium–neon gas laser (He–Ne) at 632.8 nm radiation and power levels ranging from 0.3 to 0.6 Mw.^17^

For the acquisition of spectra, the technique used was FTIR absorption spectroscopy in micro reflectance mode with accessory diamond-tipped ATR. The equipment used was a Varian—Agilent 610 FT-IR microspectrometer, coupled to a 640-IR FT-IR spectrometer (two-dimensional LN2 Ge detector), with an IR 1064 cm^–1^ laser. The spectrum acquisition window was 150 mm^2^ and the spectrum resolution was set to 4 cm^–1^ for operation at a wavelength of 4000–400 cm^–1^. The equipment was provided by the Multiuser Central Laboratory of the Federal University of ABC and the subsequent analyses were carried out using the ATR.

The spectra were obtained with 20 scans at a resolution of 4 cm^–1^, yielding an average total scan time of 20 s. All regions had the same spectrum acquisition patterns, and each sample was performed in triplicate and analyzed.

### 2.4 Data Analysis

Normality analysis was performed by using the Kolmogorov–Smirnov test for variables such as age and periodontal parameters. To evaluate the difference between groups, Student-*t* and Mann–Whitney tests were applied at a significance level of 5%. The GraphPad-Prism software version 9.5.1 was used.

All spectra were preprocessed to make them comparable for statistical analysis. The baseline was corrected by using the least-squares polynomial curve fitting method, as described by Lieber and Mahadevan-Jansen,^18^ and RStudio software. All spectra were normalized by using a mean-centering approach, which is a standard scaling method widely used in vibrational spectroscopy and metabolomic analysis.^7,15,17,19^ Principal component analysis (PCA), partial least-squares discriminant analysis (PLS-DA), and receiver operating characteristic (ROC) curve analysis were used to evaluate the diagnostic accuracy of a specific vibrational band as a biomarker. This option was implemented by using the MetaboAnalystR package running in RStudio.

In the hierarchical agglomerative cluster analysis, the samples were combined until each one belonged to a cluster. Euclidean distance was used as a measure of similarity and Pearson’s correlation coefficient as rank correlation. Clustering algorithm was average linkage and heatmap was used as a visual representation along with dendrograms. Hierarchical clustering was performed with the hclust function of the stat package in RStudio and HCA was used for inspection of clinical data.

## 3 Results

### 3.1 Demographic Data

Forty patients were evaluated and divided into two groups: control group (C) with 18 patients without peri-implant disease and the peri-implantitis group (P) with 22 patients diagnosed with peri-implantitis. Two study samples were excluded, one from the control group (C) and one from the peri-implantitis group (P), as they showed alterations in the spectral reading under the microscope and thus considered unsuitable for use in the study (i.e., they had excess water causing fluorescence effects, which affect the spectroscopic reading).

The clinical parameters were presence or absence of plaque (PI), GI, PD, and AL. According to the data obtained, the clinical parameters were higher in the peri-implantitis group compared to the control group, showing significant differences, except for PI, as no statistical difference was found between the two groups (Table 1).

**Table 1:** Description of Demographic Data and Peri-implant Parameters Evaluated

	control	peri-implantitis	*P*
*N*	17	21	
mean age	46 ± 17	53 ± 12	0.1433^a^
gender	7 M/10 F	3 M/18 F	
plaque index	0.43 ± 0.43	0.36 ± 0.42	0.7543^a^
gingival index	0.26 ± 0.35	0.53 ± 0.36	0.0241^b^,^c^
probing depth (mm)	3.41 ± 0.99	5.07 ± 2.28	0.0083^b^,^c^
attachment level (mm)	0.18 ± 0.76	5.09 ± 2.29	<0.0001^a^,^c^

a *Note*: Mann–Whitney’s test.

b Student’s *t*-test.

c Significant difference.

### 3.2 Fourier-Transform Infrared Spectroscopy

**Figure 1:**
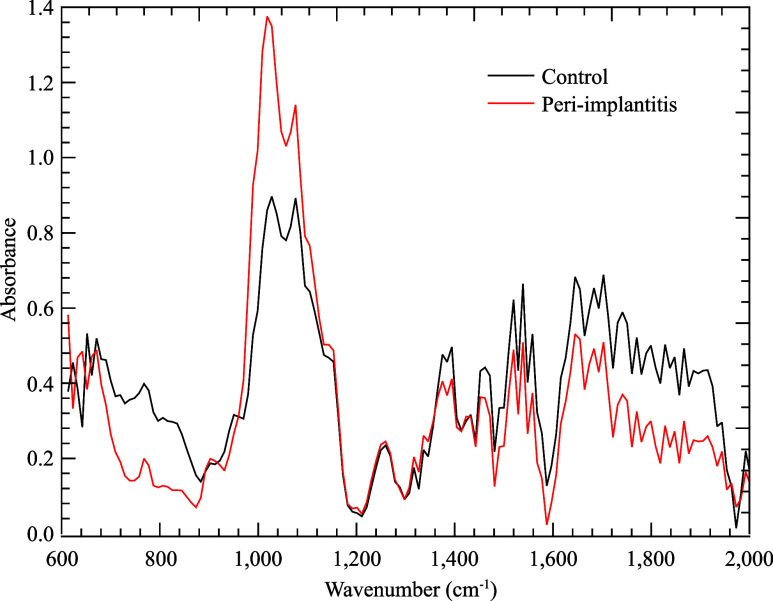
Graph representing the average spectrum for the two groups and fingerprint region ranging from 600 to 2000 cm^-1^, where the control group is represented in black and peri-implantitis group in red.

After FTIR measurement, the average raw spectrum was generated for both control (C) and peri-implantitis (P) groups, with the results shown in Figure 1. At first glance, both spectra had outstanding differences, mainly in spectral regions between 780 and 900, 1000–1100, 1390–1400, and 1700–1800 cm^–1^.

**Figure 2:**
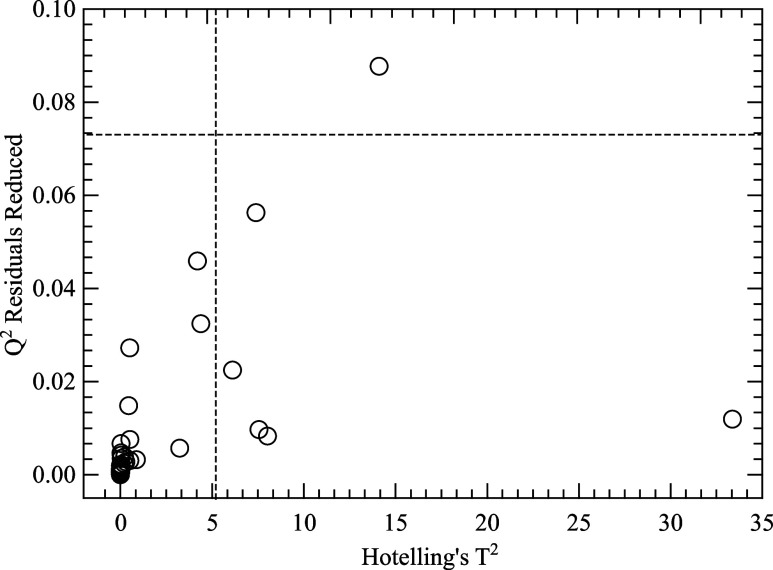
Outliers identified in the inspection of Q-residual versus Hotelling’s T^2^.

Specific bands showing intensity variations between the two groups are 1020 cm^–1^ (DNA), 1040 cm^–1^ (ribose CO stretching), 1230 cm^–1^ (asymmetric PO_2_ stretching and overlapping of protein amide III and nucleic acid phosphate vibration), 1390 cm^–1^ (carbon particle), 1430 cm^–1^ (lipids, fatty acids), 1500 cm^–1^ (in-plane CH bending of phenyl rings), 1590 cm^–1^ (phenyl *C*-ring stretching), 1650 cm^–1^ (amide I absorption), and 1700 cm^–1^ (fatty acid ester bases region).^10^. A dispersion analysis of the raw spectral data set was performed to identify anomalous spectral outliers or biased data. PCA was calculated on raw and mean-centered data, whereas Q-residual (reduced) versus Hotelling’s T^2^ was checked (Figure 2) to find outlier Q-residuals and T^2^ scores. Data outside the 95% confidence limit were considered outliers and removed from further analysis. Outliers are indicated with * on the vertical and horizontal lines, representing confidence limits of 95% (Hotelling’s) and 5% (Q-residual).

PCA was performed for classification of the FTIR spectra, and the resulting data were tested to verify the eigenvalues of the principal components (PCs) in order to determine how many PCs should be retained in the analysis. In this case, the fold was evidenced in PC 5, leading to a binary logistic regression analysis for separation of samples of Group C from those of Group P using the first five PCs.

**Figure 3:**
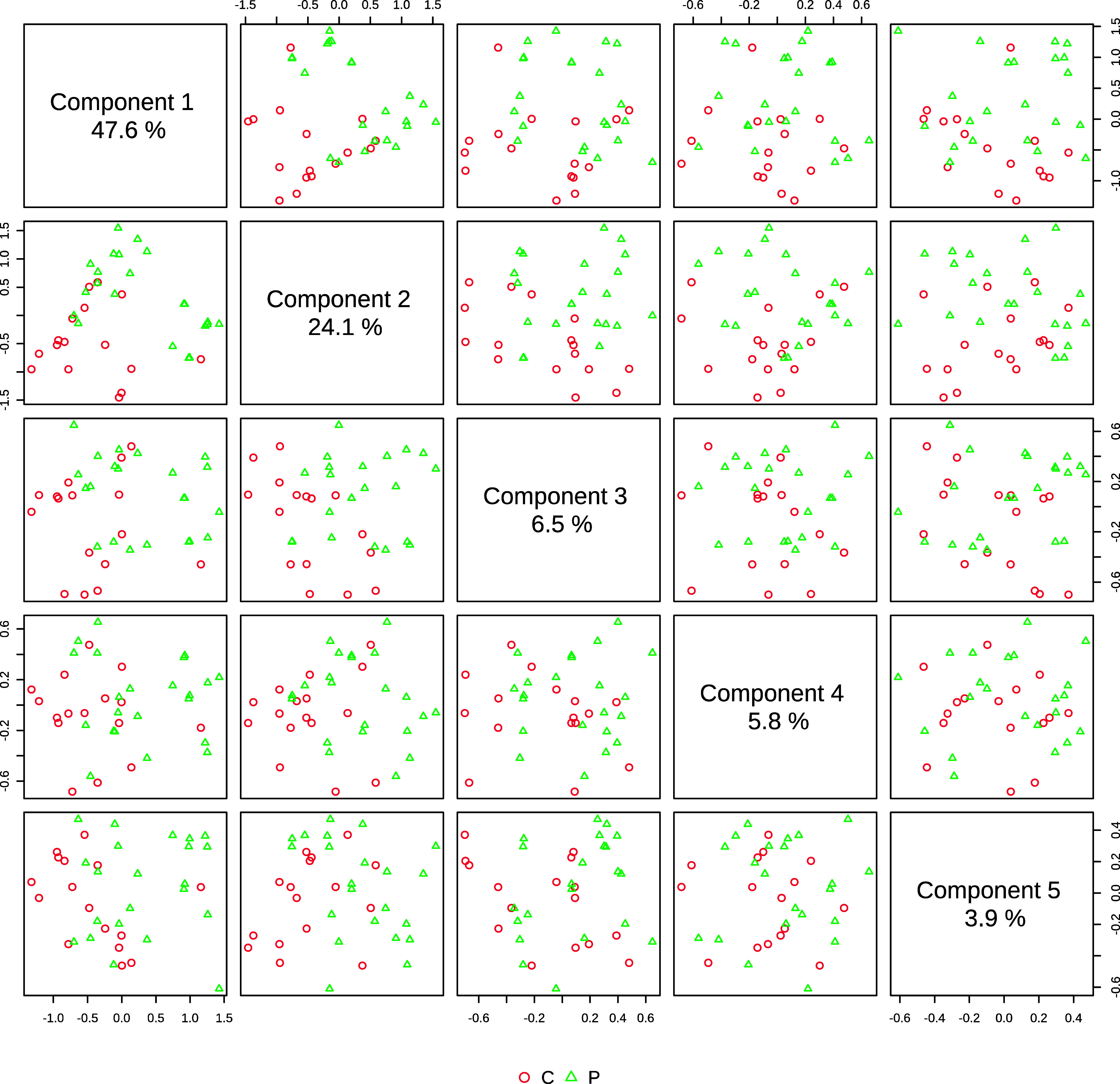
Component plot with their percentages according to the number of components.

Figure 3 shows the score pairs up to the fifth PC in which the combination included initially five components to allow for discrimination of both groups. The accuracy of the PLS-DA classification was 81% when *R*^2^ was 70%, whereas *Q*^2^ had a very close value compared to that of the combination of three components. However, the best PLS-DA classification performance was achieved with five components for the model using the groups at an accuracy of 81%.

The accuracy value with five components was higher compared with groups with fewer components, whereas the values of *Q*^2^ and *R*^2^ for the five components were also higher.

**Figure 4:**
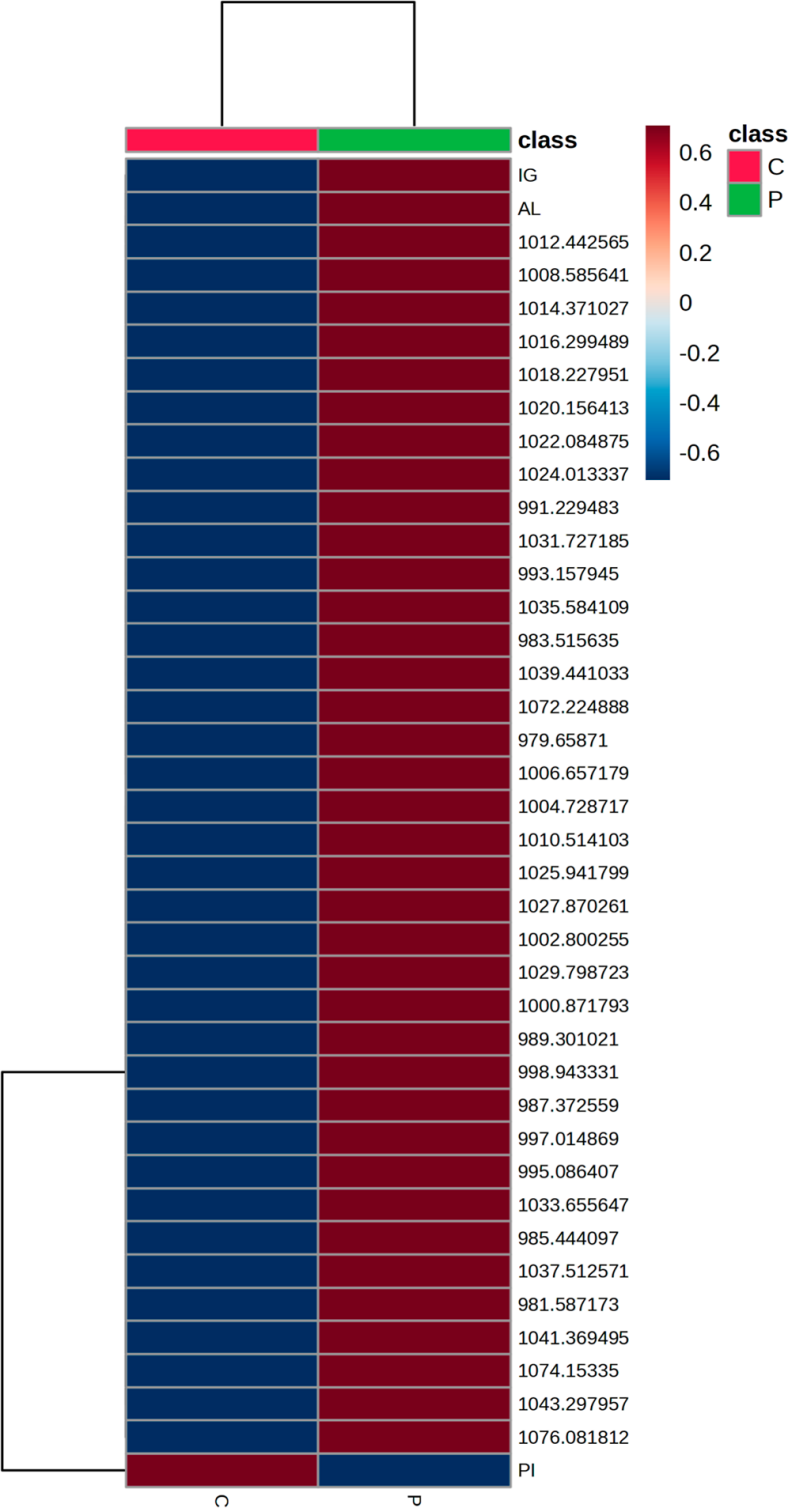
Heatmap for spectral data according to group discrimination, clinical characteristics, group separation, and main bands.

The heat maps for the general spectral data (Figure 4) revealed interesting qualitative characteristics. Two groups of patients were represented by the clusters on the left and right. It can be seen that patients with peri-implantitis had increased intensities compared to the control group, with the exception of the PI, which maintained a lower intensity scale than those of the other clinical parameters. It was also possible to evaluate the bands with the greatest alteration along with the clinical characteristics and to which group they belonged to.

A summary of assignments of the main wavenumbers and their respective vibrational modes and molecular sources making up the original spectra of saliva is presented in Table 2. It was possible to verify significant changes in the spectral bands 1558, 1698, 1733, 1749, 1760, 1772, 1835, 1845, and 1859 cm^–1^, with accuracy above 70%. These bands are related to symmetric stretching of CO–O–C along with the CO bending of the C–OH of carbohydrates and phosphate, phosphodiester stretching region, lipid stretching region, and fatty acid ester base region.

**Table 2:** Assignment of the Bands after PLS-DA Analysis

wavenumber (cm^–1^)	accuracy (%)	assignment	biomolecules	refs
1558	74	aromatic ring, amide II	tryptophan	Napomuceno et al^17^
1698	79	C=CO_2_	DNA, RNA	Dovbeshko et al^20^
1733	76	C=O in fatty acid	polysaccharides	Yoshida et al^21^
1749	80	C=O in fatty acid	polysaccharides	Shetty et al^22^
1760	75	C=CO_2_, C=O	DNA, RNA	Nogueira et al^23^
1772	70	imidazole ring	histidine	Napomuceno et al^17^
1835	73	imidazole ring	histidine	Karamancheva et al^24^
1845	76	imidazole ring	histidine	Karamancheva et al^24^
1859	70	imidazole ring	histidine	Karamancheva et al^24^

Thus, one can observe the presence of spectral markers, such as the band 1558 cm^–1^ with a signature of aromatic ring Amide II and its biomolecule being tryptophan, the band 1698 cm^–1^ with spectral signature of C=CO_2_ for thymine and purine bases as biomolecular compounds, the bands 1733 and 1749 cm^–1^ for lipid ester C=O vibration of triglycerides and polysaccharides, lipid and fatty acids representing a fatty acid ester absorption band, and the band 1760 cm^–1^ for C=CO_2_ guanine and C=O thymine with biomolecules related to DNA and RNA. The bands 1772, 1845 and 1859 cm^–1^ have histidine as signature and imidazole as biomolecule, whereas band 1835 cm^–1^ is related to NO (Table 2).

The ROC curve was used to evaluate the specificity and sensitivity of the diagnostic model, which showed a predictive value corresponding to the area under the curve, being 0.79 for the fingerprint region.

**Figure 5:**
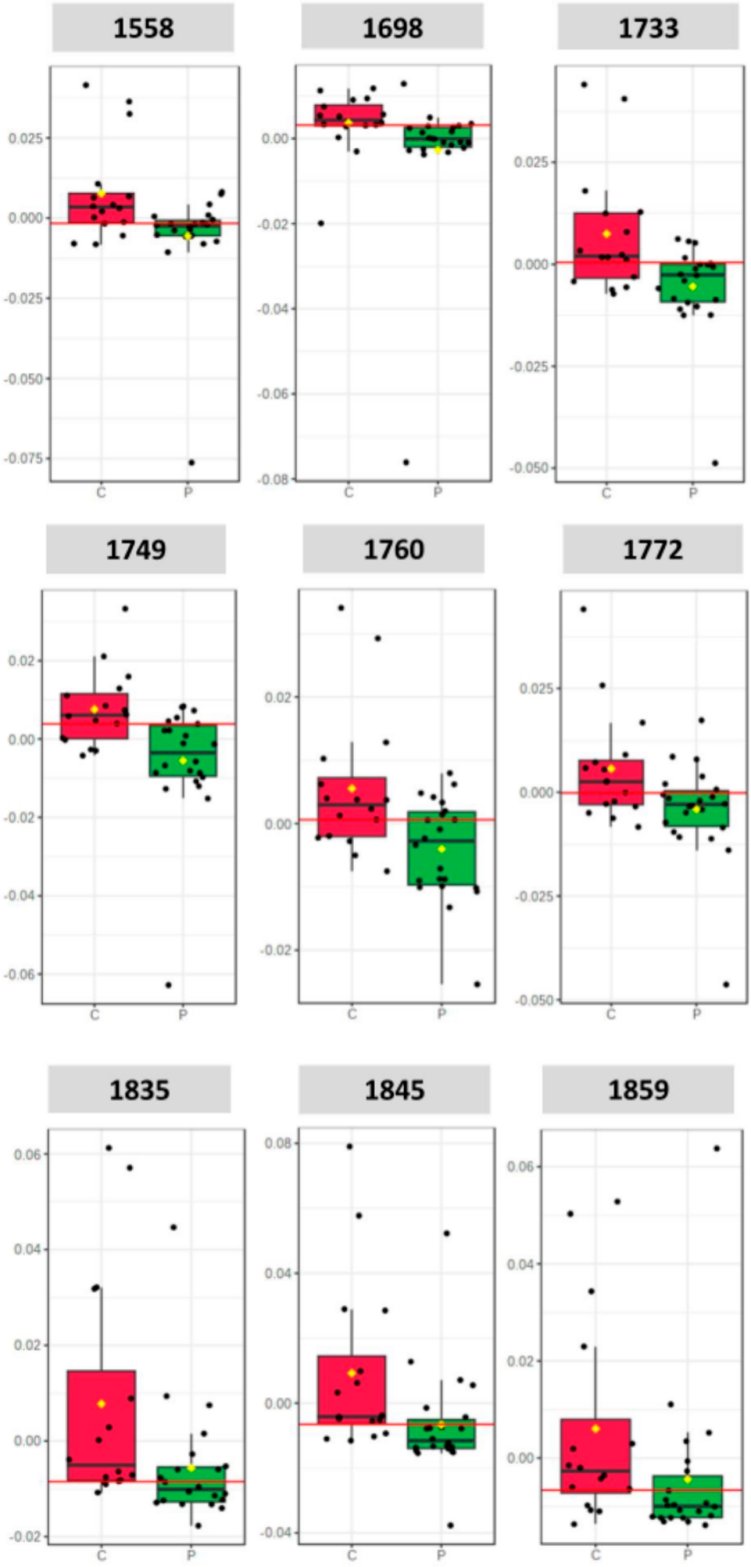
Box plot for key vibrational band candidates as spectral biomarkers showing good predictive power (AUC > 0.70).

The area under the ROC curve (AUC) was calculated for each vibrational band to further discriminate between control and *peri*-implantitis groups. Key bands with good intensity and good accuracy were considered acceptable for biomarkers (AUC > 0.70), as can be seen in Figure 5. The corresponding assignments are listed in Table 2.

The main spectra shown above were considered to have accuracy greater than 70% and their biomolecules defined, namely, histidine, fatty acids, base rings, tryptophan, and lipid esters. All were determined based on specific literature for each of them (Table 2).

## 4 Discussion

The present study evaluated the saliva of patients with and without clinical peri-implantitis by using FTIR. Although many studies have assessed the saliva of patients with periodontitis and of healthy patients for salivary component analysis with the aid of FTIR,^25,26^ this is the first one to perform an analysis of peri-implant disease.

Our results showed relevant differences between the clinically distinct groups. A total of 38 samples were evaluated using FTIR-ATR spectroscopy, known for its utility in detecting molecular-level changes. Based on this, we compared the saliva spectra of patients with and without peri-implantitis to identify potential differences. Infrared spectroscopy is increasingly recognized as an alternative modality for precise determination of lipid peroxidation in various biological samples.^27^ Increased oxidative stress results from increased production of reactive oxygen species or attenuated capacity to eliminate them, leading to tissue damage and consequently to increased lipid peroxidation. The loss of saturation during lipid peroxidation reactions was compensated by the presence of double bonds in lipid peroxidation products, such as malondialdehyde, lipid aldehydes, and alkyl radicals. It is well-recognized that lipid peroxidation increases markedly during periodontal inflammation.^28^

Tsai et al.^29^ demonstrated a positive correlation between the extent of lipid peroxidation and various clinical parameters of periodontal disease, suggesting that greater severity of periodontal tissue inflammation is associated with elevated levels of lipid peroxidation. Furthermore, oxidative stress in periodontal disease may have systemic implications, as evidenced by higher blood concentrations of lipid peroxidation in a rat model of periodontitis compared to periodontally healthy control animals.^30^

Peri-implantitis and healthy control samples showed different vibrations, such as stretching, bending, deformation, or a combination of vibrations, which are directly related to the molecular structure of the constituent biomolecules.^26^ This finding provides complementary information to support the possible use of saliva as a less invasive chairside diagnostic method, in addition to the periodontal clinical examination with a probe. Peri-implantitis is a chronic inflammatory disease leading to loss of dental implants,^31,32^ which is demonstrated by changes in nucleic acids directly involved in the synthesis processes of cell and collagen fibers.

Peak assignments showed more variation in the distribution of DNA (DNA) and lipids, such as in the bands 1698, 1733, 1749, and 1760 cm^–1^, which was also reported by Shetty et al.,^22^ and corroborates the findings that saliva can reliably identify portions of DNA and lipids. According to Nepomuceno et al.,^17^ band 1698 cm^–1^ is attributed to guanine C=O, which makes up DNA and is increased in carcinogenesis processes. Similar to the study by Xiang et al.,^33^ band 1713 cm^–1^ is represented as a DNA strand with base pairs, where increased DNA concentrations in gingival crevicular fluid samples were observed in patients with periodontal changes compared to healthy ones. Increased DNA content in samples of inflammatory gingival crevicular fluid may suggest a provocative condition during an inflammatory condition, with active leukocytes, bacteria, and sloughed epithelial cells in the gingival crevicular fluid samples.

In the study by Fujii et al.,^34^ the salivas of healthy and periodontitis patients were evaluated with the aid of FTIR for band differentiation of the disease. They found that the most characteristic peak was between the bands 1800 and 1500 cm^–1^, where the spectral characteristics are denoted by the carbonyl ester C=O absorption band at 1738 cm^–1^. Amide I band, which is represented by C=O stretching, was observed between the bands 1700 and 1600 cm^–1^, whereas the amide II band was observed between the bands 1600 and 1500 cm^–1^. Part of these findings is consistent with the data obtained in the present study.

The properties of saliva in patients with periodontal disease differ from those in normal patients as it is recognized that Gram-negative bacteria, such as Porphyromonas gingivalis, Aggregatibacter actinomycetemcomitans, and Prevotella intermedia, spread into the gingival sulcus pockets. This corroborates our findings on bands related to peri-implantitis as well as those reported by Shibli et al.,^11^ Das et al.,.^35^

Peri-implantitis is a significant contributor to medium- and long-term implant failure. Identifying biomarkers associated with this condition is crucial for the development of effective prevention and therapeutic strategies. These biomarkers hold promise as noninvasive diagnostic tools for early detection, disease monitoring, and personalized management of peri-implantitis.^36^ The most extensively studied biomarkers for peri-implantitis include proinflammatory cytokines IL-1β, IL-6, IL-12, IL-17, and TNF-α; anti-inflammatory cytokines IL-4 and IL-10; osteoclastogenic markers RANK, RANKL, and OPG; antioxidant proteins: urate, malondialdehyde, ascorbate, and myeloperoxidase; and chemokine: IL-8. Research has demonstrated that peri-implantitis is associated with elevated salivary levels of IL-1β, IL-6, and IL-10. These interleukins have been proposed as potential biomarkers for the early diagnosis and monitoring of this condition. Additionally, salivary levels of IL-8 and IL-12 have been found to be significantly higher in patients with peri-implantitis compared with those with peri-implant mucositis. Furthermore, TNF-α levels are reported to be elevated in peri-implantitis patients relative to healthy individuals.^37,38^

There was a difference in clinical data between the two groups, except for PI, which was not a decisive factor in peri-implantitis as it was clinically observed and confirmed through the heatmap. The study by Shibli et al.^11^ identified that the main pathogens found in the peri-implantitis group were *P. gingivalis*, *T. forsythia*, *T. denticola*, and *P. nigrescens*, warning that the supragingival biofilm of diseased implants can serve as a reservoir for pathogenic species. This contributes to the reinfection of already treated areas,^39^ who highlighted the virulence of *P. gingivalis* associated with implants, which reinforces our findings regarding the PI as it would not be an isolated factor for the occurrence of peri-implantitis. In addition, Pallos et al.^13^ confirmed previous data by using microbiome analysis in saliva samples. There were differences between peri-implant groups (diseased × healthy) regarding the alpha and beta diversity, suggesting that saliva samples could also be an important tool to identify oral infections related to peri-implantitis.

Furthermore, Sugimoto et al.^5^ used saliva as a biofluid to evaluate specific biomarkers for diseases such as breast, pancreatic, and oral cancer, as well as periodontal problems by analyzing metabolites involved in each disease. A total of 215 subjects were evaluated, including 69 with oral cancer, 30 with breast cancer, 18 with pancreatic issues, 11 with periodontal problems, and 87 healthy controls. They found 57 metabolites to discriminate individuals with periodontal disease, and of these, 27 were reported as important candidate biomarkers for periodontal disease, including tryptophan, histidine, carnitine, alanine, glutamic acid, pipecolic acid, and polyamine threonine. Spectral analysis showed that the main changes in inflammatory activity affected biomarkers such as tryptophan and histidine. The study by Napomuceno et al.^17^ reported an association with tryptophan by evaluating biomarkers of kidney disease, demonstrating its effectiveness as an indicator of heart disease development in patients with kidney problems. Moreover, tryptophan is a precursor of serotonin and melanin. Compared with the study in question, tryptophan and histidine were also important biomolecules serving as biomarkers for patients with peri-implantitis, thus corroborating the studies mentioned-above studies.

In the study by Kuboniwa et al.,^25^ the prediction of periodontal inflammation was assessed through analysis of salivary metabolic profile in which it was possible to affirm that histidine concentration was increased in subgingival areas. They found eight metabolites identified as potential indicators of periodontal inflammation, with the combination of cadaverine, 5-oxoproline, and histidine presenting satisfactory accuracy (AUC = 0.881). This aligns with our findings on histidine alterations in peri-implant regions as FTIR analysis indicates that histidine is a good biomarker for periodontitis (Simsek et al., 2016).^40^ This was also demonstrated by two more studies on oral cancer and oral squamous cell carcinoma through the evaluation of metabolites.^41,42^ Histidine, an imidazole ring, was also found in the bands 1772, 1845, and 1859 cm^–1^, thus being an important biomarker in the present study.

In this present study, the boxplot graph showed that the largest spectral variations between peri-implantitis and control samples in the fingerprint region were related to histidine, indicating its effectiveness in protecting inflamed tissue due to the imidazole ring’s ability to eliminate reactive oxygen species generated by cells during the acute inflammatory response. When administered in therapeutic doses, histidine can inhibit cytokines and growth factors involved in cell and tissue damage.^39^

Finally, our model using five PCs for diagnosis was the one that best identified the variations found among the samples with the highest accuracy. According to das Chagas e Silva de Carvalho et al.,^43^ it is important to verify PCs as PCA proved to be significant for up to 10 PCs. The FTIR spectroscopy technique using high wavenumber fingerprint regions identified a relationship (0.81) between control and peri-implant groups through PCA. This indicates that it can be used as a complementary examination to clinical and radiographic evaluations.

Heatmaps also showed two well-defined clusters between the two groups. It was possible to verify the relationship of the clinical data with the most relevant bands (Table 2). The intensity of each data point and its cluster was evaluated by using a color scale.

The present study demonstrated that saliva can be an easy and expensive way to detect peri-implantitis, at least in severe cases. Further studies should be performed to identify possible local risk factors regarding some clusters and the proteomic profile before and after peri-implantitis treatment.

## 5 Conclusions

Within the limits of this case-control study, we were able to probe and identify relevant molecular signatures in the saliva of peri-implant patients compared to the control group. As salivary profiles with relevant alterations are correlated to the peri-implant disease, we concluded that FTIR is a viable and successful technique to investigate the saliva of patients with severe peri-implantitis.

## Data Availability

The data that support the findings of this study are available at the European Zenodo repository by the link: 10.5281/zenodo.14540221.
